# An Efficient Phosphorus Adsorbent Prepared from Calcium/Iron-Rich Storm Sewer Sludge: Performance and Mechanism

**DOI:** 10.3390/molecules31142534

**Published:** 2026-07-21

**Authors:** Yan Wu, Jinhui Chen, Luyue Zhang, Yi Chen, Haiyan Ye, Qingguo Wang

**Affiliations:** 1 School of Architecture and Civil Engineering, Xihua University, Chengdu 610039, China; wuy@mail.xhu.edu.cn (Y.W.);; 2 Engineering Technology Research Center for Pollution Control and Ecological Restoration of Impaired Rivers and Lakes, Xihua University, Chengdu 610065, China; 3 College of Architecture and Environment, Sichuan University, Chengdu 610065, China

**Keywords:** urban storm sewer, dredged storm sewer sludge, pyrolysis, phosphate adsorption, adsorption mechanism

## Abstract

Calcium- and iron-rich sludge from urban storm sewer is an ideal source of phosphorus adsorbents. This study used urban storm sewer sludge to prepare phosphorus adsorbents via pyrolysis. Comparing adsorbents prepared under different conditions, the optimal material was produced at 800 °C in a nitrogen atmosphere and was named 800N. Analyses were conducted using scanning electron microscopy, X-ray diffraction, Fourier-transform infrared spectroscopy, Brunauer–Emmett–Teller surface area analysis, and X-ray photoelectron spectroscopy. The results showed that 800N is rich in carbon, calcium, and iron. The adsorbent has a pore volume of 0.013 cm^3^/g and a specific surface area of 3.566 m^2^/g. Adsorption performance was most effective at a pH of 8, achieving an adsorption capacity (q_e_) of 19.32 mg/g and a removal rate of 87.75%. Kinetic and thermodynamic studies revealed that the adsorption of phosphorus by the adsorbent conforms to the pseudo-second-order kinetic model and the Langmuir isotherm model. Based on the characterization results, it can be reasonably inferred that the primary mechanisms involved in phosphorus adsorption by the adsorbent are inner-sphere complexation, ligand exchange, and chemical precipitation. This study offers a novel solution for mitigating phosphorus pollution and promoting the resource utilization of urban storm sewer sludge.

## 1. Introduction

Phosphorus is one of the primary indicators for controlling water pollution. Once the phosphorus concentration in water reaches 100 μg/L, eutrophication is likely to occur [1]. Excessive phosphorus discharge has led to an intensification of eutrophication in water bodies, becoming one of the main issues confronting global surface waters today [2,3]. Each country has set stringent standards for phosphorus emissions [1]. Yet the problem of phosphorus pollution continues [4]. The use of phosphate fertilizer continues to rise to meet the demands of population growth and regional development [3]. The water bodies remain impacted by phosphorus pollution from sources including agriculture, livestock, poultry farming, urban households and aquaculture [5]. The ongoing increase in phosphorus pollution underscores the need for effective measures to mitigate environmental phosphorus contamination [6].

Phosphorus removal methods encompass physic-chemical and biological processes [7]. The efficacy of biological phosphorus removal is contingent upon a complex interplay of environmental and operational parameters, such as dissolved oxygen concentrations, ambient temperature, biochemical oxygen demand (BOD), and the nature of organic substrates [8]. Physic-chemical methods include precipitation, ion exchange, and adsorption. Precipitation and ion exchange require the addition of chemical reagents or specialized equipment, which can be inconvenient [9,10]. Chemical adsorption is widely utilized for phosphorus removal due to its simplicity, high efficiency, ease of recovery, and availability of suitable adsorbent materials [11,12].

Recently, materials have been designed specifically as phosphorus adsorbents [13]. To further reduce costs, the focus has shifted towards utilizing industrial by-products and waste materials as raw materials for developing phosphorus adsorbents [14]. Among these, sludge, a waste product from water treatment industries, contains significant amounts of organic matter and can serve as a precursor for high-carbon materials suitable for adsorbent preparation [15,16]. In recent years, numerous papers have emerged on sewage sludge biochar (SBC). Pyrolysis can transform sludge into high-value-added carbonaceous materials [17,18].

Sludge from wastewater treatment plants has been prepared as adsorbents for pollutant removal [19,20]. This is achieved through the pyrolysis of sludge under air or inert conditions [21,22], which significantly reduces its volume, eliminates pathogens and parasites, and completely decomposes organic residues [23]. Compared to traditional adsorbents such as activated carbon, zeolites, and graphene, adsorbents derived from sludge demonstrate high-efficiency adsorption performance and provide cost benefits [24]. Besides wastewater plant sludge, a substantial amount of drainage channel sludge also requires appropriate disposal annually [24]. For instance, in Shanghai, the production of drainage channel sludge from pipeline maintenance reached 210,500 m^3^ in 2023 [25].

Drainage channel sludge, obtained from cleaning drainage networks after pipe maintenance, dredging, and excavation, consists of a mixture of water and mineral particles with an organic carbon content ranging from 9% to 23% [26]. Compared to municipal wastewater plant sludge, drainage channel sludge contains relatively lower levels of organic matter, with its inorganic fraction primarily composed of inactive components such as SiO_2_, CaCO_3_, and Fe_2_O_3_, whose proportions vary across different functional areas [27,28]. Carbon-based adsorbents containing or loaded with Ca, Mg, Al, and Fe have shown significant effectiveness in phosphorus adsorption from water bodies [29,30,31,32]. Various carbon-based phosphorus adsorbents have been developed in existing studies, including activated carbon, agricultural residue-derived biochar, metal-modified biochar, and municipal sewage sludge-derived biochar. Compared with commercial activated carbon, biochar produced from stormwater sludge has prominent economic advantages [33]. Agricultural biomass-derived biochar has intrinsically low Ca, Fe and Al contents, thus requiring costly post-synthetic modification that elevates process complexity and production costs [34]. Meanwhile, the large-scale application of municipal sewage sludge biochar is severely constrained by high heavy metal loads and associated environmental risks [35]. In contrast, stormwater sludge is widely available with lower heavy metal contamination, and it is naturally enriched with Ca-, Fe- and Si-bearing minerals originating from urban sediments and construction materials, which can provide abundant active sites for phosphate adsorption and precipitation. Inspired by existing research, adsorbent materials prepared from the pyrolysis of drainage channel sludge hold promising prospects for phosphorus adsorption in water bodies.

Accordingly, storm sewer dredged sludge was selected as the raw material in this study. Sludge-based adsorbents were fabricated via pyrolysis under nitrogen atmosphere and oxidative heat treatment under air atmosphere, respectively, at temperatures of 600, 700, 800 and 900 °C. The primary objectives of this work are as follows: (1) To investigate the effects of treatment temperature and atmosphere (inert pyrolysis vs. oxidative heat treatment) on phosphate adsorption performance, and identify the optimal preparation conditions for the sludge-derived adsorbents. (2) To systematically characterize the as-prepared adsorbents using SEM-EDS, BET, XRD, FTIR and XPS techniques, and elucidate their key physicochemical properties including surface morphology, elemental distribution, pore structure, crystalline phase composition and surface functional groups. (3) To evaluate the impacts of critical operating parameters such as adsorbent dosage, solution pH and coexisting ions on the phosphate removal performance of the adsorbents. (4) To reveal the underlying phosphate removal mechanisms of the dredged sludge-derived adsorbent.

## 2. Results and Discussion

### 2.1. The Impact of Pyrolysis Temperature and Atmosphere on Phosphate Adsorption

**Figure 1:**
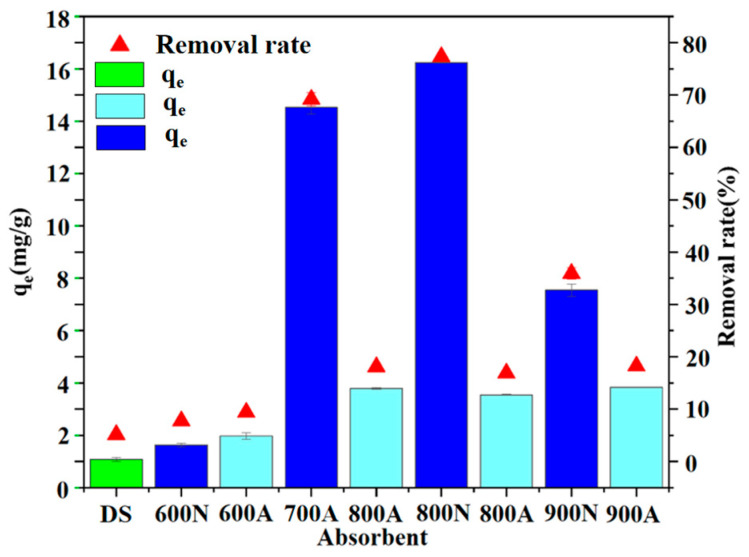
Phosphorus adsorption by different adsorbents (C_0_ = 20 mg/L, dosage = 1 g/L, pH = 6, t = 24 h, T = 300 K).

**Figure 2:**
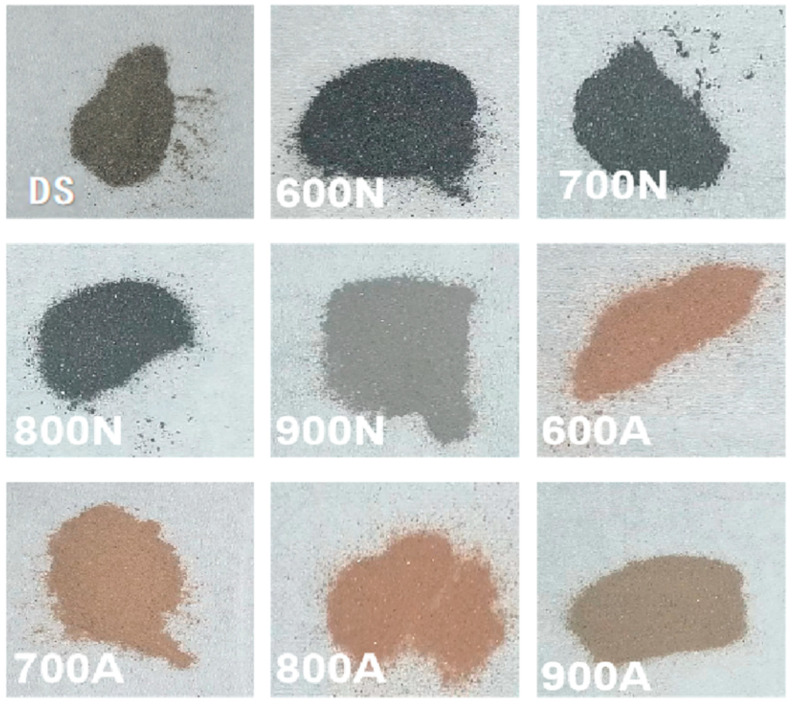
Pictures of adsorbents under different atmospheres and temperatures.

The adsorption capacity (*q_e_*) and removal rate (%) of the adsorbents for phosphate adsorption under different pyrolysis conditions are shown in Figure 1. Significant differences in adsorption capacity and removal rate were observed among the various adsorbents. For dredged storm sewer sludge (DS), the adsorption capacity and removal rate were 1.09 mg/g and 5.17%, respectively. Under air conditions, the maximum phosphate removal rate reached 18.24% at 900 °C, with an adsorption capacity (*q_e_*) of 3.83 mg/g, under a nitrogen atmosphere. The removal rate increased from 7.76% at 600 °C to 69.17% at 700 °C, peaking at 77.31% at 800 °C, while dropping to 35.93% at 900 °C. The maximum adsorption capacity (*q_e_*) of 16.24 mg/g was achieved at 800 °C. Under an inert atmosphere, the adsorbent prepared at 600 °C turned black (Figure 2), which was consistent with the trends observed by Jiang et al. regarding cellulose pyrolysis products [36]. This color change indicates a higher degree of carbonization in the adsorbent. Compared to other conventional adsorbents [37,38]. When the pyrolysis temperature increases to 900 °C, the material’s color becomes lighter and adsorption efficiency decreases. At 900 °C, the residual organic matter undergoes complete and deep thermal decomposition. Part of the formed carbonaceous substances was further gasified. This gasification occurs through thermal cracking and secondary reactions with pyrolysis volatiles. It leads to a significant reduction in solid-phase carbon content [39]. Meanwhile, the high temperature of 900 °C promotes the crystallization and surface sintering of inorganic minerals. This reveals the light-colored inorganic matrix originally covered by the black biochar [40]. Moreover, at 900 °C, sintering causes the collapse of the pore structure in the carbon fraction. It also decreases the specific surface area [41]. 800N was selected as the adsorbent for subsequent phosphate adsorption experiments because it exhibited the highest adsorption performance under this preparation condition.

### 2.2. Characterization of Pyrolysis Sludge

#### 2.2.1. Surface Morphology and Elemental Composition of Adsorbents

The surface micromorphology of the optimal 800N adsorbent was characterized via scanning electron microscopy (SEM) at gradient magnifications, and its elemental composition was analyzed by energy-dispersive X-ray spectroscopy (EDS).

**Figure 3:**
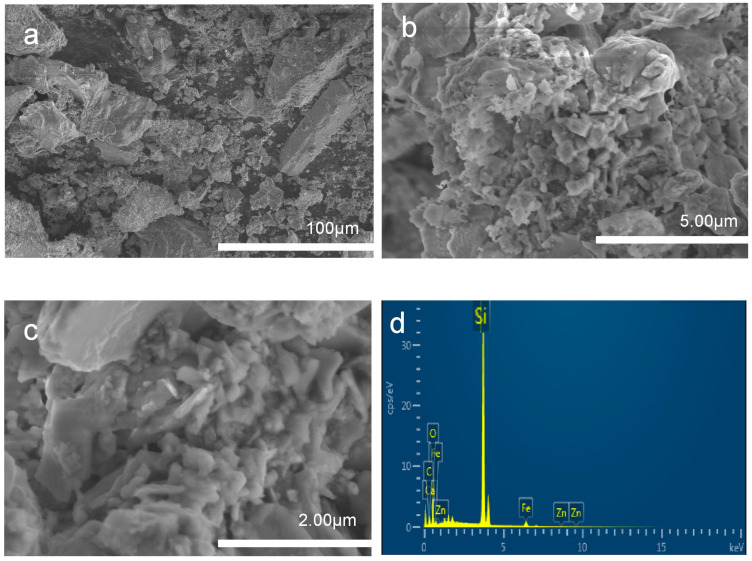
SEM-EDS maps of 800N (**a**–**c**): SEM, (**d**): EDS.

As presented in the low-magnification SEM image (Figure 3a, scale bar = 100 μm), the 800N sample was composed of irregular fragmented particles with distinct angular edges and a wide particle size distribution. The particles aggregate randomly, forming abundant intergranular gaps and macroporous structures between adjacent fragments. At medium magnification (Figure 3b), the surface of bulk particles exhibits a rough and uneven texture, covered with densely attached fine granular precipitates. Further high-magnification observation (Figure 3c) reveals that the surface was constructed by stacked microscale aggregates with abundant surface reliefs and pore structures, presenting a highly heterogeneous and coarse topography. This hierarchical rough surface and well-developed porous structure effectively enlarge the contact area between the adsorbent and phosphate solution, providing sufficient active sites for phosphate retention and facilitating the adsorption process [42].

The EDS spectrum of 800N was shown in Figure 3d. The results demonstrate that the sludge-derived adsorbent mainly consists of silicon, oxygen, calcium, iron and carbon, with trace amounts of zinc (Zn) detected. The predominance of Si, Ca and Fe elements confirms that the 800N sample was rich in inorganic mineral components, which is consistent with the high-ash characteristics of storm sewer dredged sludge. Detailed quantitative data of elemental weight fraction and atomic fraction are summarized in Table S1.

#### 2.2.2. Surface Phase Structure of Adsorbents

**Figure 4:**
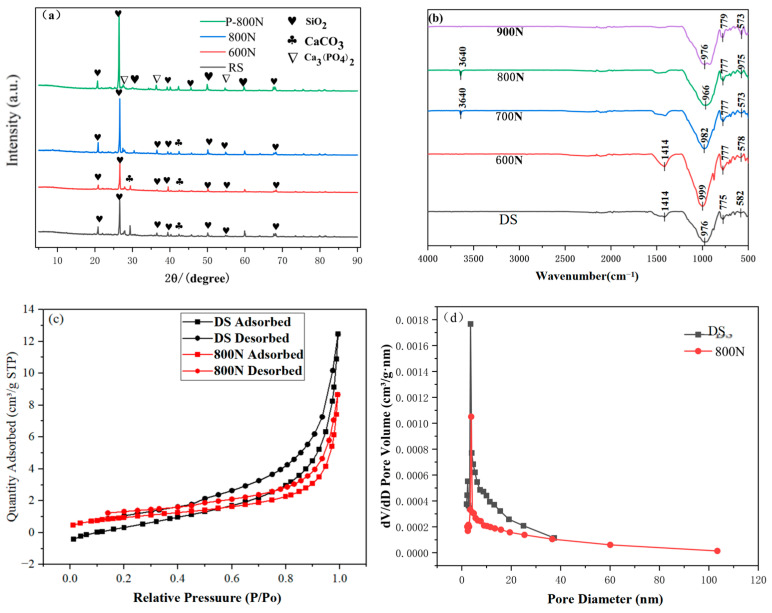
(**a**) XRD patterns of DS, 600N, 800N, and P-800N; (**b**) FTIR patterns of DS, 600N, 700N, 800N, and 900N; (**c**) adsorption–desorption isotherms of DS and 800N; (**d**) pore size distribution of DS and 800N.

The crystalline phase compositions of samples DS, 600N, 800N, and P-800N (800N after phosphate adsorption) are presented in Figure 4a. Phase identification was performed by matching diffraction patterns with standard PDF cards (Figures S2–S5), and the results reveal that the 800N’s dominant crystalline phases include SiO_2_, FePO_4_, and Ca_3_(PO_4_)_2_, which was consistent with the EDS elemental analysis results. Characteristic diffraction peaks of SiO_2_ are detected at 2θ = 20.7°, 26.4°, 36.4°, 39.0°, 45.7°, 49.9°, 54.7°, 59.8°, and 68.0°.

The peaks assigned to CaCO_3_ (29.5°, 39.5°, and 42.5°) gradually weaken in intensity with increasing pyrolysis temperature. This trend arises from the thermal decomposition of calcium carbonate at elevated temperatures, which generates reactive calcium oxide and other active metal oxide species [29,43]. These in situ formed metal oxides are highly beneficial for phosphate removal, as they provide abundant active adsorption sites and participate in surface precipitation reactions [44,45]. After phosphate adsorption onto 800N, new distinct diffraction peaks emerge at 39.3° and 41.1°, which are attributed to the formation of Ca_3_(PO_4_)_2_ precipitates, directly confirming chemical precipitation of calcium-bearing minerals as a core phosphate removal pathway.

The FTIR spectra of RS, 600N, 700N, 800N, and 900N are shown in Figure 4b. The characteristic peaks of different functional groups were observed. The absorption peak observed at 3640 cm^−1^ was attributed to the stretching vibration of O–H bonds within hydroxyl groups. [46,47]. The adsorbent’s hydroxyl groups can exchange ions with phosphate ions [48]. Additionally, these hydroxyl groups and other functional groups become protonated under low pH conditions, acquiring a positive charge [31]. The charge variations on the surface of the adsorbent can influence phosphate adsorption through electrostatic forces. The absorption peak at 3640 cm^−1^, observed exclusively in samples pyrolyzed at 700–800 °C, can be assigned to the stretching vibration of structural hydroxyl groups in Ca(OH)_2_ [49]. With rising pyrolysis temperature, CaCO_3_ decomposes progressively into reactive CaO; during the cooling process, partial CaO reacts with ambient moisture to form Ca(OH)_2_, which accounts for the detection of the 3640 cm^−1^ peak in the 700–800 °C samples. At 900 °C, however, CaO undergoes further solid-phase reactions with silicon-bearing constituents to form more stable calcium silicate minerals [50]. This reduces the content of rehydratable free CaO and leads to the re-disappearance of the 3640 cm^−1^ peak. The peak at 1414 cm^−1^ was attributed to the asymmetric stretching vibration of CO_3_^2−^. Its intensity decreases markedly and even vanishes at temperatures above 600 °C, indicating that elevated temperature promotes carbonate decomposition and the formation of CaO. The generated CaO then participates in further mineral phase reconstruction to form more stable calcium silicate or calcium phosphate phases [51]. The peak at 1414 cm^−1^ corresponds to C–N stretching vibrations. Peaks in the range of 966–999 cm^−1^ are associated with out-of-plane bending vibrations of C–C bonds [47], which overlap with the stretching vibration absorption peaks of Si–O–Si. The peak at 777 cm^−1^ can be attributed to both in-plane and out-of-plane bending vibrations of C–H bonds [52,53]. The absorption peak observed at 578 cm^−1^ was characteristic of Fe–O bonds [54].

#### 2.2.3. Surface Area and Pore Structure of Adsorbents

Figure 4c,d present the adsorption–desorption isotherms and pore size distributions of DS and 800N. Both samples exhibit Type II isotherms with Type H3 hysteresis loops, indicating irregular pore structures in each sample. As shown in Table S2, the specific surface area of 800N was 3.566 m^2^/g, significantly higher than that of DS at 1.350 m^2^/g. The larger specific surface area of the adsorbent provides more adsorption sites, thereby enhancing phosphate adsorption efficiency [46]. Compared to DS, the pore volume of 800N decreased from 0.016 cm^3^/g to 0.013 cm^3^/g, likely due to the decomposition of organic and inorganic components in the sludge during pyrolysis. Meanwhile, the average pore diameter increased from 10.38 nm for DS to 15.79 nm for 800N. Pores within the range of 2–50 nm are classified as mesopores. Thus, both DS and 800N can be categorized as mesoporous adsorbents. Notably, the pore size of 800N was significantly larger compared to DS. The increase in pore size suggests that a substantial amount of gas was released during the pyrolysis process, forming larger pores in 800N [55]. Increased porosity, specific surface area, and pore number are advantageous for enhancing the adsorbent’s phosphorus adsorption capacity [56].

#### 2.2.4. The Surface Chemistry Analysis

**Figure 5:**
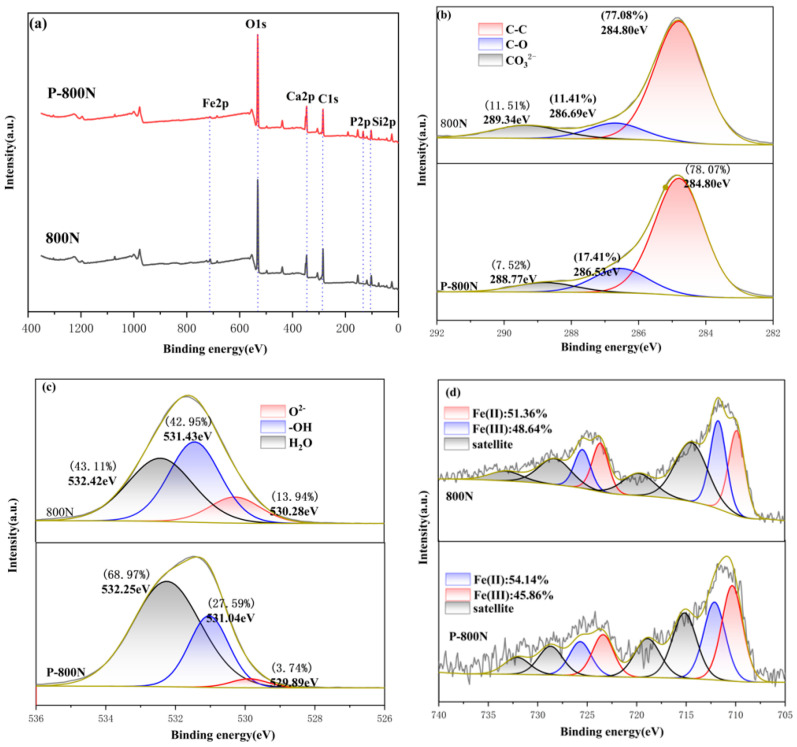
XPS spectra of 800N before and after phosphorus adsorption: (**a**) full spectra of 800N and P-800N; (**b**) C 1s spectra of 800N and P-800N; (**c**) O 1s spectra of 800N and P-800N; (**d**) Fe 2p spectra of 800N and P-800N.

To further investigate the phosphate adsorption mechanism of the prepared 800N, X-ray photoelectron spectroscopy (XPS) analysis was conducted. The XPS survey spectra of 800N and P-800N revealed that the primary surface constituents of 800N are Fe, O, Ca, C, and Si. The P 2p spectrum centered at 133.2 eV confirmed the successful adsorption of phosphates [57]. To delve deeper into the removal mechanism of phosphates, high-resolution C 1s spectra were obtained (Figure 5b). After phosphate adsorption by 800N, the proportion of CO_3_^2−^ decreased. This difference indicates the transformation of CaCO_3_ on the 800N surface into Ca_3_(PO_4_)_2_ [58,59]. The O 1s peak of 800N consists of three distinct peaks located at 530.28 eV, 531.43 eV, and 532.42 eV (Figure 5c), corresponding to lattice oxygen (O^2−^), hydroxyl groups (–OH), and adsorbed water (H_2_O), respectively. After phosphate adsorption, the relative peak area assigned to hydroxyl groups decreased, which may suggest the possible involvement of surface hydroxyl groups in the adsorption process. In addition, all three O 1s peaks shifted toward lower binding energies, indicating that oxygen-containing surface species participated in phosphate adsorption [58,60].

In the Fe 2p spectra (Figure 5d), satellite peaks indicative of Fe oxides were observed in both the Fe 2p_1_/_2_ and Fe 2p_3_/_2_ spectra. Peaks at 709.84 eV and 711.71 eV correspond to Fe 2p_3_/_2_, where the lower energy peak (709.84 eV) represents Fe^2+^ and the higher energy peak (711.71 eV) represents Fe^3+^. Similarly, peaks at 723.14 eV and 724.87 eV correspond to Fe 2p_1_/_2_, with Fe^2+^ at 723.14 eV and Fe^3+^ at 724.87 eV. After phosphate adsorption, the binding energies of the Fe 2p_1_/_2_ and Fe 2p_3_/_2_ shifted to higher values, indicating iron’s involvement in the phosphate reaction and its role in phosphorus adsorption [61,62]. On 800N, the ratios of Fe(II) and Fe(III) were 51.36% and 48.64%, respectively, whereas on P-800N, these ratios changed to 54.14% and 45.86%. The Fe(III) content reduction also indicates its involvement in the phosphate reaction process [63].

### 2.3. Batch Adsorption of Phosphate by Pyrolyzed Sludge

#### 2.3.1. Effect of Adsorbent Dose

**Figure 6:**
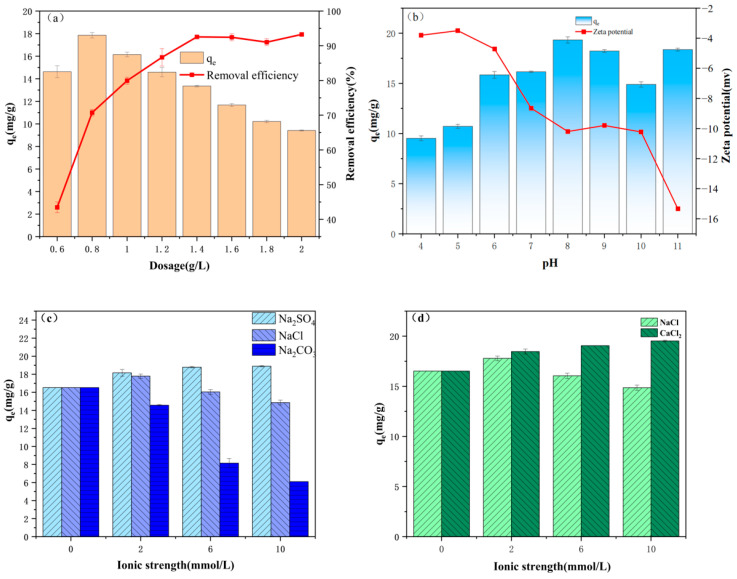
(**a**) Effect of varying adsorbent dosages on phosphate adsorption. (**b**) Influence of different pH levels on phosphate adsorption efficiency. (**c**) Impact of anions on phosphate adsorption. (**d**) Influence of cations on phosphate adsorption.

To assess the phosphate adsorption performance of 800N, we examined how different dosages affected phosphate removal efficiency. As illustrated in Figure 6a, increasing the dosage of 800N within a certain range provided more active sites for adsorption, significantly boosting phosphate removal. However, exceeding an optimal dosage resulted in unused adsorption sites, diminishing returns, and inefficient use of resources. At a dosage of 1 g/L, the removal rate peaked at 80%, with an adsorption capacity of 16.1 mg/g. We chose a dosage of 1 g/L for further experiments to balance effectiveness and cost efficiency.

#### 2.3.2. Effect of pH

The pH of the solution significantly influences the adsorption performance of phosphate, as it determines both the form of phosphate present and the surface charge of the adsorbent [46]. As shown in Figure 6a, the adsorption capacity increases steadily with pH from 4 to 8, reaching its peak at pH 8, where the adsorption amount (q_e_) and removal rate are 19.32 mg/g and 87.75%, respectively. However, as the pH increases from 8 to 10, the adsorption capacity decreases and then rises again from pH 10 to 11. At pH 11, the removal rate was 83.99%. This behavior was primarily attributed to the significant effect of initial pH on the surface charge of 800N. The relationship between initial pH and zeta potential is illustrated in Figure 6b. The adsorption capacity increased steadily as the initial pH ranged from 4 to 8, reaching a maximum at pH 8. Table S3 summarizes the predominant forms of phosphate ions under different pH conditions. This increase can be attributed to reduced competition between H^+^ ions and phosphate ions for active adsorption sites, as well as to the gradual conversion of phosphate species from H_2_PO_4_^−^ to HPO_4_^2−^, the latter having a stronger affinity for calcium-containing active sites. When the pH increased from 8 to 10, the adsorption capacity decreased slightly. At this stage, the increased concentration of OH^−^ competed with phosphate for the active sites on the surface [64]. However, when the pH further increased to 11, calcium released from the adsorbent promoted the formation of insoluble calcium phosphate precipitates, partially compensating for the reduced adsorption and shifting the dominant phosphorus removal mechanism from surface adsorption to calcium-induced precipitation [65,66]. Consequently, the phosphorus removal mechanism gradually shifts from surface adsorption to calcium-induced precipitation at high pH, which explains the observed decrease followed by a recovery in adsorption performance.

#### 2.3.3. Effect of Coexisting Ions

The effects of coexisting anions and cations on the phosphate adsorption performance of 800N are illustrated in Figure 6c,d. As the concentration of Na^+^ increases, it exhibits a slight inhibitory effect on the equilibrium adsorption capacity (q_e_). Conversely, increased Ca^2+^ concentration significantly enhances phosphate adsorption, likely due to the formation of calcium phosphate complexes [31]. The influence of cations on phosphate adsorption efficiency follows the order: Ca^2+^ > Na^+^.

The hydrolysis of bicarbonate ions (HCO_3_^−^) increases the solution pH, deprotonating surface hydroxyl groups on the adsorbent. This increases electrostatic repulsion between the adsorbent and phosphate anions, reducing phosphate adsorption [31,67]. In contrast, increasing the SO_4_^2−^ concentration improves phosphate adsorption by 800N. In acidic solutions, low sulfate concentrations promote calcium release, enhancing phosphate removal [68]. Chloride ions (Cl^−^) exhibit a minimal inhibitory effect on q_e_, but this influence is negligible. Regarding anions, their impact on phosphate adsorption efficiency follows the sequence: HCO_3_^−^ > SO_4_^2−^ > Cl^−^.

Coexisting anions compete for available adsorption sites on the 800N surface, potentially inhibiting phosphate adsorption [54,69]. However, the experimental results demonstrate that the overall impact of coexisting ions on phosphorus adsorption is minimal. The lack of significant competitive adsorption indicates that 800N possesses strong selectivity for phosphate ions. These findings support the practical application of 800N as an effective phosphorus adsorbent, highlighting its robust performance even in the presence of various coexisting ions.

#### 2.3.4. Adsorption Kinetics

**Figure 7:**
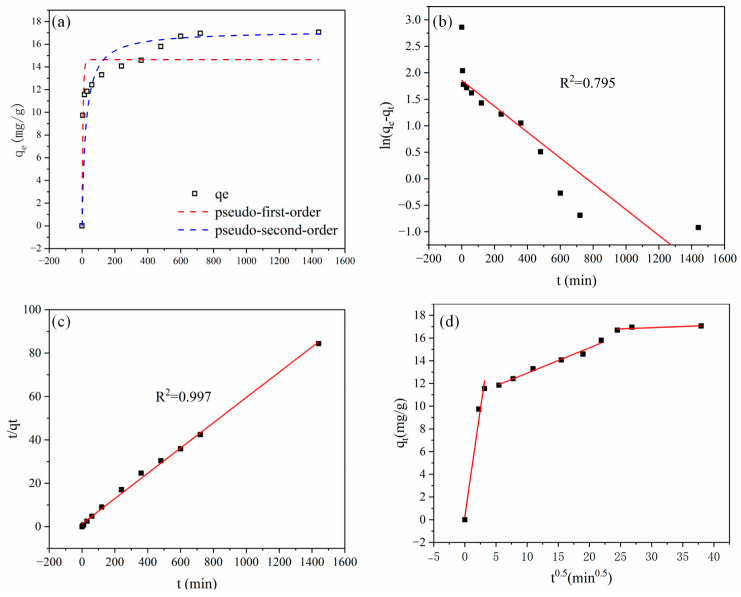
Images of 800N adsorption kinetics: (**a**) phosphate adsorption over time; (**b**) pseudo-first-order model fitted adsorption kinetics; (**c**) pseudo-second-order model fitted adsorption kinetics; (**d**) intra-particle diffusion model fitted adsorption kinetics.

As shown in Figure 7a, phosphate was rapidly immobilized, with nearly 70% of the adsorption occurring within the first 90 min. The adsorption process reached a relatively stable equilibrium state after 1440 min. The pseudo-first-order, pseudo-second-order, and intraparticle diffusion models were used to describe the adsorption behavior of phosphate on 800N. The results indicated that the pseudo-second-order kinetic model had a higher R^2^ value (0.997) than the pseudo-first-order model (R^2^ = 0.795), suggesting that the pseudo-second-order model better describes the adsorption behavior. The theoretical q_e_ values obtained from the pseudo-second-order model also closely matched the experimental adsorption values. These findings indicate that chemical adsorption dominates the adsorption process.

The intraparticle diffusion model was applied to elucidate the adsorption limiting mechanisms (Figure 7d). The plot shows three distinct stages: a rapid surface adsorption stage, a gradual adsorption stage, and a final equilibrium stage. During the rapid surface adsorption stage, phosphate ions quickly traverse the boundary layer surrounding the adsorbent. They are rapidly adsorbed onto the surface, with this phase primarily limited by film diffusion [70]. In the gradual adsorption stage, intraparticle diffusion appears to influence the kinetic behavior of adsorption. The fact that the fitted curve does not pass through the origin indicates that adsorption in this stage is not solely controlled by intraparticle diffusion but rather by the combined effects of intraparticle and film diffusion. In the final equilibrium stage, the slope of the curve reaches equilibrium due to low phosphate concentrations and a scarcity of active sites on the adsorbent surface, resulting in a very slow adsorption rate. Tables S2 and S3 summarize the corresponding parameters.

#### 2.3.5. Adsorption Isotherms

**Figure 8:**
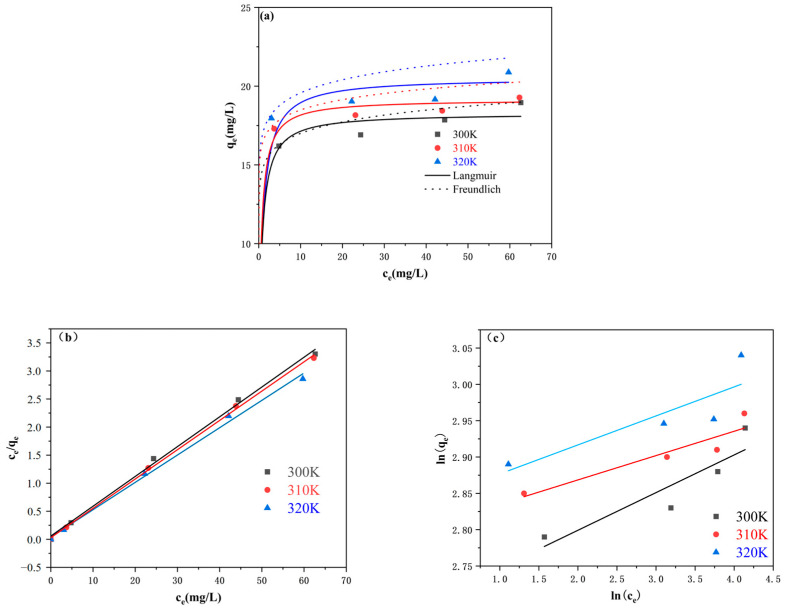
Images of 800N isothermal adsorption experiments: (**a**) phosphate adsorption as a function of initial concentration; (**b**) adsorption isotherm simulated by Langmuir model; (**c**) adsorption isotherm simulated by Freundlich model.

The Langmuir and Freundlich isotherm models were selected to evaluate the adsorption mechanism of phosphate on the prepared 800N. As shown in Figure 8, the Langmuir model exhibited significantly higher R^2^ values (0.997 at 300 K, 0.999 at 310 K, and 0.996 at 320 K) compared to the Freundlich model (R^2^ = 0.828, 0.869, and 0.736). This indicates that the Langmuir model adequately describes the phosphate adsorption isotherms on 800N, suggesting that the adsorption primarily occurs as a monolayer process. With increasing temperature from 300 K to 320 K, the maximum adsorption capacity (q_m_) and the Freundlich constant (K_F_) increased. This indicates that elevated temperatures enhance phosphate adsorption, suggesting that the adsorption process is endothermic [71]. The positive Langmuir constant (K_L_) values indicate the spontaneity of the adsorption process [72]. A higher value indicates greater spontaneity. Table S4 summarizes the corresponding parameters.

### 2.4. Phosphate Removal Mechanism

Adsorption isotherm and kinetic analyses revealed that chemical interactions primarily drive phosphate adsorption by 800N [73]. The adsorption data fit well with the Langmuir model, suggesting phosphate adsorption on 800N occurs as a monolayer process.

Inner-sphere complexation is one of the primary mechanisms for phosphorus adsorption by 800N. XPS analysis comparing 800N and P-800N revealed that carbon-containing functional groups participate in phosphorus adsorption through complexation [59]. Hydroxyl groups within the adsorbent can form hydrogen bonds or coordination bonds with phosphate ions, facilitating phosphorus adsorption [31]. Additionally, phosphate can form new complexes with Ca [74] and Fe [75]. Outer-sphere complexation relies on electrostatic forces, and coexisting anions significantly influence its effectiveness [31,76]. In coexistence ion adsorption experiments, the adsorption performance of 800N was not significantly influenced by coexisting anions. This indicates that the primary mechanism for phosphorus adsorption by 800N is inner-sphere complexation.

Ligand exchange is one of the primary mechanisms for phosphorus adsorption by 800N. After phosphorus adsorption, the peak areas of –OH and CO_3_^2−^ in 800N decrease, indicating that these groups are replaced by phosphate through ligand exchange [31,54]. SEM-EDS analysis shows that 800N is rich in Ca on its surface. Calcium can attach to the adsorbent as cations and undergo ligand exchange with phosphate ions, forming complexes that deposit on the pyrolyzed sludge surface, thereby removing phosphate [77,78]. XPS analysis indicates that iron participates in the phosphorus reaction (Figure 5d) [63,64]. The Fe on the surface of 800N can form Fe–O–P groups through ligand exchange [75].

Surface precipitation is considered a major phosphate removal pathway for 800N, particularly because calcium released from the adsorbent can react with phosphate to form insoluble calcium phosphate phases under alkaline conditions. X-ray diffraction (XRD) analysis (Figure 4a) reveals that the major components of 800N are silicon dioxide (SiO_2_), calcium carbonate (CaCO_3_), and calcium oxide (CaO). After phosphate removal by 800N, the appearance of new diffraction peaks at 39.3° and 41.1° indicates the formation of calcium phosphate (Ca_3_(PO_4_)_2_). Calcium ions released from the surface of 800N can react with phosphate to form insoluble calcium phosphate phases, including Ca_3_(PO_4_)_2_, CaHPO_4_ and Ca_5_(PO_4_)_3_(OH) [31,46]. Therefore, phosphorus removal by 800N involves not only surface adsorption but also Ca-induced surface precipitation, which is expected to become increasingly important under alkaline conditions. Additionally, phosphate can precipitate with iron ions (Fe^2+^ and Fe^3+^) present on the surface of 800N, forming compounds like iron(II) phosphate (Fe_3_(PO_4_)_2_) and iron(III) phosphate (FePO_4_) [79,80]. Surface precipitation thus plays a critical role in the phosphorus removal capacity of 800N.

The zeta potential of 800N remained negative throughout the investigated pH range (Figure 6b), indicating that electrostatic attraction between the negatively charged adsorbent surface and phosphate anions is unlikely to be the dominant driving force for phosphate removal. Despite the unfavorable electrostatic conditions, 800N maintained a high phosphate removal capacity, suggesting that chemical interactions play a predominant role.

**Figure 9:**
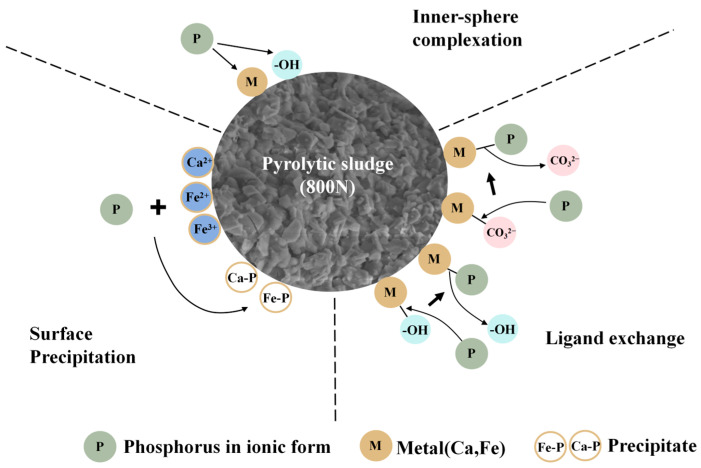
Phosphate removal mechanism of phosphate by 800N.

The prepared adsorbent (800N) is characterized as a mesoporous material with a complex pore structure, as evidenced by scanning electron microscopy (SEM) and Brunauer–Emmett–Teller (BET) analyses (Table S2). This unique pore structure increases the specific surface area and provides additional active sites. Pores in the 2–50 nm range facilitate organic phosphorus pollutants’ adsorption [81]. This mesoporous nature enhances the accessibility of active sites within the adsorbent, thereby improving its overall efficiency in phosphorus removal. Comprehensive characterization results demonstrate that phosphate removal by 800N involves multiple mechanisms, including inner-sphere complexation, ligand exchange, and Ca-induced surface precipitation. Among these, chemical precipitation is likely to make a substantial contribution because of the high calcium content of 800N and the formation of calcium phosphate identified by XRD, although the individual contributions of each mechanism cannot be quantitatively distinguished based on the current data. The primary mechanisms include inner-sphere complexation, ligand exchange, and chemical precipitation (Figure 9).

## 3. Materials and Methods

### 3.1. Materials

The dredged storm sewer sludge used in this experiment was collected from the storm sewer in the High-tech Zone of Chengdu City, Sichuan Province of China. A picture of the on-site sampling process is shown in Figure S1. All reagents used in this experiment were of analytical grade. Potassium dihydrogen phosphate (KH_2_PO_4_) was sourced from Chengdu Kelong Chemicals Co., Ltd. (Chengdu, China). KH_2_PO_4_ was dissolved in deionized water to prepare a phosphate stock solution with a 1000 mg/L concentration. This stock solution was then diluted to prepare the working solutions for subsequent batch experiments.

### 3.2. Adsorbent Preparation

The dredged sludge was sampled in June 2023. The samples were ground and then dried in an oven at 60 °C for 48 h. The dried product was sieved through a 100-mesh sieve, stored in a desiccator, and properly labeled and sealed. Subsequently, the dredged sludge was subjected to pyrolysis and oxidative heat treatment in a tube furnace under two different atmospheric conditions (continuous nitrogen and continuous air). Under a nitrogen atmosphere, the heating rate was 5 °C/min. The samples were held at four different temperatures (600 °C, 700 °C, 800 °C, and 900 °C) for 2 h each [33,34], and the pyrolysis products were named 600N, 700N, 800N, and 900N, respectively. Under continuous air flow conditions, sludge calcined ash was obtained using the same heating protocol. The products of the oxidative heat treatment were designated as 600A, 700A, 800A, and 900A, respectively.

Under continuous air conditions, using the same heating protocol, the oxidation heat treatment products were designated as 600A, 700A, 800A, and 900A, respectively.

### 3.3. Characterization and Analytical Method

Scanning electron microscopy (SEM) coupled with energy-dispersive X-ray spectroscopy (EDS) was performed using a JSM-7500F field-emission scanning electron microscope (JEOL Ltd., Akishima, Tokyo, Japan) to observe the surface morphology of the adsorbents at multiple magnifications and analyze the elemental distribution in selected sample regions.

The specific surface area and pore structure of the adsorbents were determined via nitrogen adsorption–desorption measurements at 77 K on an ASAP-2460 physical adsorption analyzer (Micromeritics Instrument Corp., Norcross, GA, USA). The specific surface area was calculated using the Brunauer–Emmett–Teller (BET) method, and the pore size distribution was derived from the Barrett–Joyner–Halenda (BJH) model.

X-ray diffraction (XRD) analysis was carried out on an EMPYREAN X-ray diffractometer (Malvern Panalytical, Malvern, UK) with Cu Kα radiation (λ = 0.15406 nm) at an operating voltage of 40 kV. The test was conducted in continuous scanning mode over a 2θ range of 5–80°, with a scan rate of 0.026° s^−1^.

Fourier transform infrared (FTIR) spectroscopy was conducted on an INVENIO spectrometer (Bruker Corp., Billerica, MA, USA) to identify the characteristic functional groups on the surface of the as-prepared adsorbents, with a scanning wavenumber range of 500–4000 cm^−1^.

X-ray photoelectron spectroscopy (XPS) was performed using an AXIS Ultra DLD spectrometer (Kratos Analytical Ltd., Manchester, UK) to characterize the surface chemical states of the adsorbents before and after phosphorus adsorption. Both full survey spectra and high-resolution spectra of target elements were collected, and all binding energy values were calibrated against the C 1s peak at 284.8 eV.

### 3.4. Batch Adsorption Experiments

Batch experiments were conducted to investigate the adsorption behavior of the adsorbent towards phosphate. In each experiment, 100 mL of a phosphate solution (pH 6, concentration 20 mg/L) was added to a 150 mL conical flask containing the adsorbent. The dosage of the adsorbent was 1 g/L. The flasks were placed in a shaker and agitated at 180 rpm and 300 K for 24 h. After shaking, the supernatant was filtered through a 0.45-μm filter membrane, and the phosphate concentration was determined using the molybdate spectrophotometric method. The initial pH of the phosphate solution was adjusted using 0.1 mol/L NaOH and HCl solutions. Each test was performed in duplicate, and the average values were used for analysis.

Adsorption efficiency is calculated by Equation (1).

(1) $$q_{e}=\frac{V(C_{0}-C_{t})}{m}$$

Note: *q_e_* is the phosphorus removal capacity per unit mass of adsorbent at equilibrium (mg/g); *C*_0_ and *C_e_* are the initial phosphorus concentration and the phosphorus concentration at equilibrium (mg/L), respectively; *V* is the volume of the phosphorus-containing solution (L); *m* is the mass of the adsorbent added (g).

To determine the kinetics of the adsorption process, residual phosphate concentrations were measured at different time intervals ranging from 5 min to 1440 min. Three typical kinetic models were used for simulation: pseudo-first-order kinetics, pseudo-second-order kinetics, and the intraparticle diffusion model. The equations for these models are provided in Text S1.

The adsorption isotherm process was investigated using initial phosphate concentrations ranging from 20 mg/L to 80 mg/L and temperatures at 300 K, 310 K, and 320 K. The experimental isotherm data were fitted to the Langmuir and Freundlich models, detailed in Text S2.

Additionally, batch adsorption experiments were conducted to study the effects of dosage, pH, and coexisting ions on the adsorbent’s adsorption performance. These factors included the dosage of the adsorbent (ranging from 0.6 g/L to 2.0 g/L), the initial pH of the solution (ranging from 4 to 11), and coexisting ions (HCO_3_^−^, Cl^−^, SO_4_^2−^, Na^+^, Ca^2+^) at concentrations of 2 mmol/L, 6 mmol/L, and 10 mmol/L.

## 4. Conclusions

In this study, a highly efficient and practical adsorbent was prepared from storm sewer sludge collected in Chengdu using pyrolysis carbonization. This adsorbent effectively removes phosphorus. By investigating the effects of pyrolysis temperature and atmosphere on phosphate removal, it was determined that the adsorbent prepared at 800 °C under a nitrogen atmosphere exhibited the best performance for phosphate adsorption. This optimal performance can be attributed to the adsorbent’s composition (800N), which contains C, O, Si, Ca, and partial Fe elements. Additionally, 800N features a large pore volume, high specific surface area, and an effective pore size distribution. The primary adsorption mechanisms are concluded to include inner-sphere complexation, ligand exchange, and chemical precipitation. Therefore, this study provides an effective strategy for the resource recovery of storm sewer sludge and the facile acquisition of phosphate adsorbents. It contributes to the realization of treating waste with waste and supports green development.

### Environmental Implication

Regular dredging of sludge from sewers is essential for maintaining urban pipeline systems. The unique composition of dredged storm sewer sludge, distinct from typical sludge, influences reaction conditions and mechanisms, thus limiting pyrolysis research. This study prepared a mesoporous pyrolyzed sludge adsorbent with active adsorption sites by pyrolyzing dredged storm sewer sludge. The performance and mechanisms of phosphorus removal were studied, with the primary mechanisms identified as inner-sphere complexation, ligand exchange, and chemical precipitation. These findings offer an effective solution for dredged storm sewer sludge disposal and resource utilization, achieving the goal of waste-to-waste treatment.

## Data Availability

The original contributions presented in this study are included in the article/Supplementary Materials. Further inquiries can be directed to the corresponding author.

## Supplementary Materials

The following supporting information can be downloaded at: https://www.mdpi.com/article/10.3390/molecules31142534/s1. Reference [82] is cited in the Supplementary Materials.
