# Supplementary material for: An Efficient Phosphorus Adsorbent Prepared from Calcium/Iron-Rich Storm Sewer Sludge: Performance and Mechanism
Source: Molecules. 2026 Jul 21;31(14):2534. doi: 10.3390/molecules31142534 (PMC13414603; doi:10.3390/molecules31142534)
Supplement: Supplementary file 1 [file molecules-31-02534-s001.zip › molecules-4397877-supplementary.pdf]

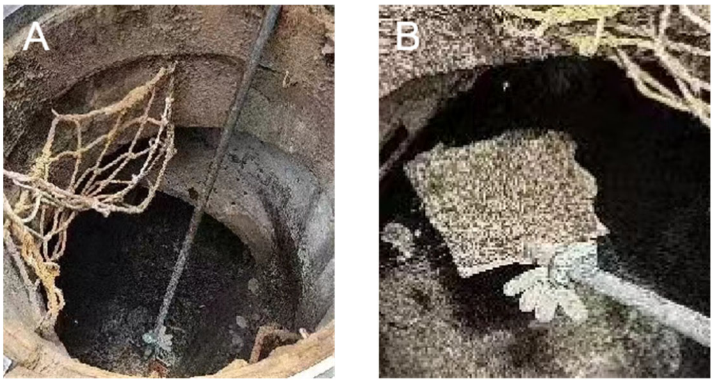

Figure S1. Photos from the sampling site

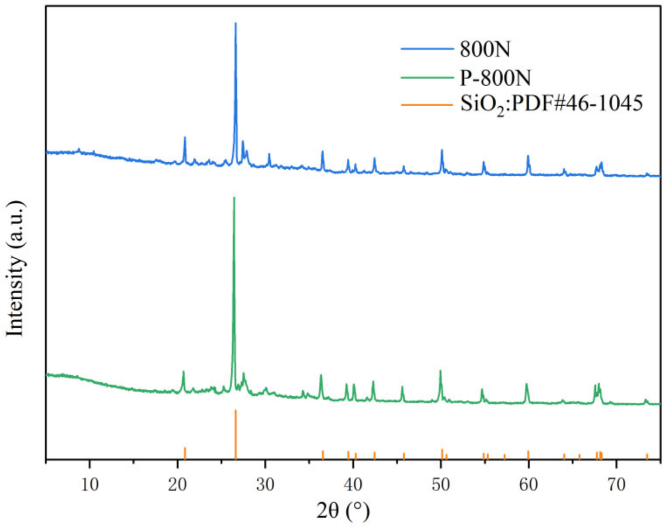

Figure S2. Comparison of adsorbent materials with SiO_2 standard cards.

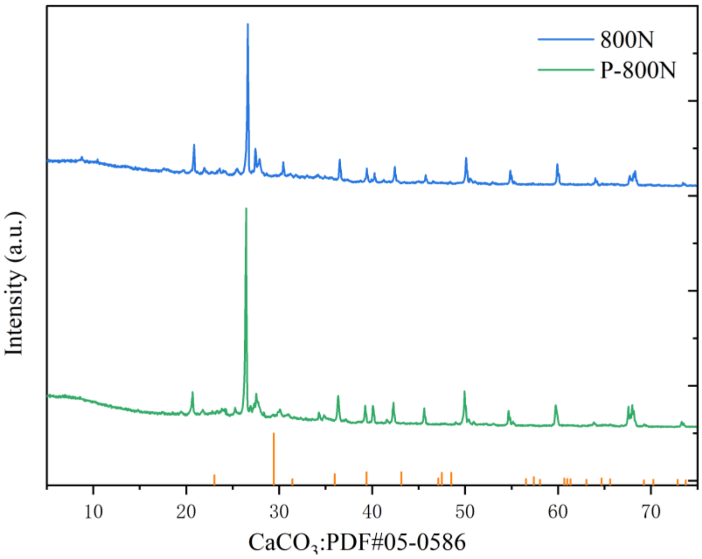

Figure S3. Comparison of adsorbent materials with $CaCO_3$ standard cards.

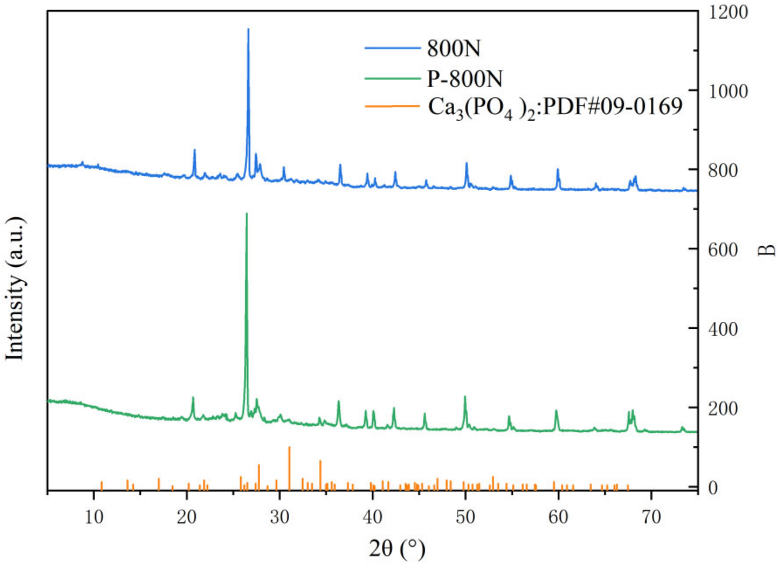

Figure S4. Comparison of adsorbent materials with $Ca_3(PO_4)_2$ standard cards.

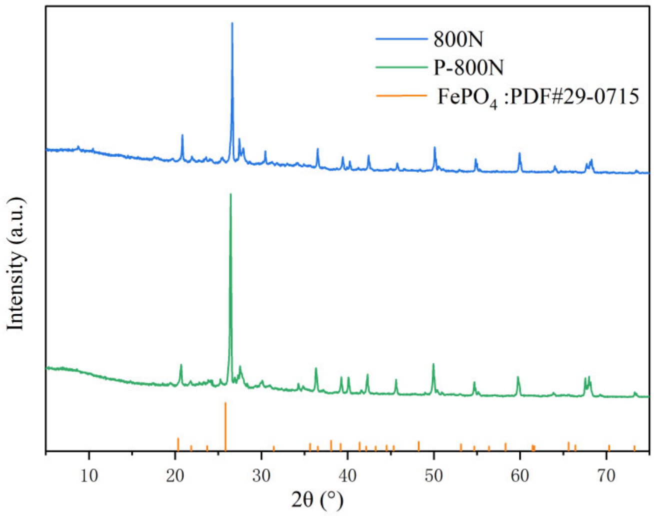

Figure S5. Comparison of adsorbent materials with $FePO_4$ standard cards.

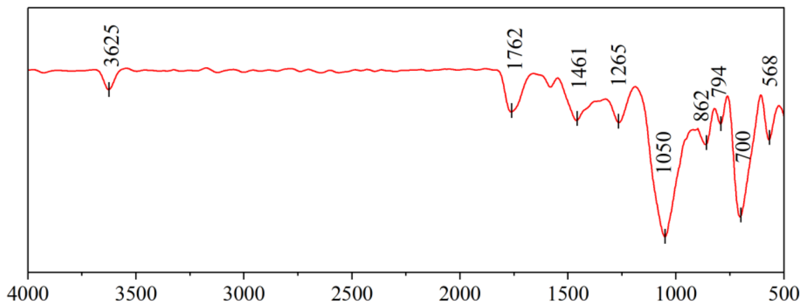

Figure S6. FTIR spectrum of 800N after adsorption

Table S1 List of elementals

Elementals	Percentage of weight%
C	15.27
O	50.81
Si	12.08
Ca	16.52
Fe	5.28
Zn	0.03
Overall amount	100.00

Table S2 Specific surface area and pore properties of adsorbents

Samples	Specific surface area(m^2/g)	Pore volume(cm^3/g)	Apertures(nm)
RS	1.3502	0.015855	10.3804
800N	3.5658	0.012644	15.7904

Table S3 The main form of PO_4^{3-}

Phosphate ions exist in the form	PH range
H_3PO_4	<2.13
$H_2PO_4^-$	2.13-7.13
HPO_4^{2-}	7.13-12.32
PO_4^{3-}	>12.13

Table S4 800N isothermal adsorption model fitting parameters

Langmuir model				Freundlich model			
K	q_m(mg/g)	K_L(L/mg)	R^2	K	K_F （L/mg)	1/n	R^2
300	17.73	0.19	0.581	300	14.56	0.04	0.996
310	18.82	0.33	0.732	310	16.49	0.03	0.999
320	19.91	0.35	0.460	320	16.93	0.06	0.997

Table S5 800N adsorption kinetic model fitting parameters

Pseudo-first-order			Pseudo-second-order		
$K_1/(g\cdot mg^{-1}\cdot min^{-1})$	q_e/(mg/g)	R^2	$K_2/(g\cdot mg^{-1}\cdot min^{-1})$	q_e/(mg/g)	R^2
0.17	14.64	0.847	0.002	17.18	0.997

Table S6 800N particle internal diffusion model fitting parameters

Phase I			Phase 2			Phase 3		
k_1 (g/mg/min)	C_1 (mg/g)	R_1^2	K_2 (g/mg/min)	C_2 (mg/g)	R_2^2	K_3 (g/mg/min)	C_3 (mg/g)	R_3^2
3.78	0.28	0.980	0.22	10.68	0.981	0.021	16.28	0.671

Text S1 Adsorption kinetic modeling

Quasi-primary kinetics, quasi-secondary kinetics and intraparticle diffusion models were used to fit the adsorption process in the adsorption kinetics. The formulations of the three equations are shown in (S1), (S2) and (S3), respectively.

$$\ln(q_e - q_t) = \ln q_e - k_1 t \quad \text{(S1)}$$

$$\frac{t}{q_t} = \frac{1}{k_2 q_e^{2}} + \frac{t}{q_e} \quad \text{(S2)}$$

$$q_t = k_p t^{0.5} + C \quad \text{(S3)}$$

Where t (min) is the adsorption time, q_e (mg/g) is the equilibrium adsorption amount, q_t (mg/g) is the adsorption amount at time t, k_1 (min^{-1}), k_2 (min^{-1}), and k_p ($(g/(mg \cdot min)^{-1}$) are the quasi-primary, quasi-secondary, and intraparticle diffusion modeling constants, respectively, and C is a constant.

Text S2 Isothermal adsorption model

Langmuir and Freundlich's models were used in isothermal adsorption modelling to fit the adsorption process. The formulations of the two equations are shown in (S4) and (S5), respectively.

$$c_e / q_e = q_e / q_m + 1/(K_L q_m) \quad \text{(S4)}$$

$$q_e = K_F + C_e^{1/n} \quad \text{(S5)}$$

Where q_m (mg/g) is the theoretical maximum adsorption capacity, n is Freundlich adsorption constant (dimensionless), C_e is the concentration of the solute in the solution at adsorption equilibrium, dimensionless; K_F ($(mg/g)\cdot(L/mg)^n$) [1] and K_L (L/mg) are Langmuir and Freundlich's adsorption constants, respectively, and n is an empirical constant.

[1] FOO K Y, HAMEED B H. Insights into the modeling of adsorption isotherm systems [J]. Chem Eng J, 2010, 156(1): 2-10.
